# Novel Use of Mercury

**Published:** 1890-03-01

**Authors:** 


### NOVEL USE OF MERCURY.

Some American physicians are now advising the use of flannel impregnated with finely-divided metallic mercury, when it is desirable to introduce this agent into the system. Soft thick flannel is selected, and soaked for several hours in an alkaline bath, consisting of a pound of carbonate of soda dissolved in four pints of water. The flannel after one rinsing in cold water and wringing is ready for a mercurial solution made as follows: In a large porcelain dish place several pounds of quicksilver—enough to be sure of excess—and add several pints of cold dilute nitric acid. When reaction has ceased the dish is to be set aside in the open air for twenty-four hours. Should no crystals of nitrate appear, some concentrated nitric acid must be added. When crystals begin to form, it is a sign that the solution is sufficiently concentrated. The flannel, still moist and alkaline, should then be dipped into it. After four or five hours it is taken out, the solution allowed to drain off, and the flannel soaked for a few minutes in a third mixture, made with one pint of ammonia and two of water. As soon as the flannel has turned a greyish black colour, it needs only drying in the open air with protection from direct sunshine, to be ready for use. Flannel thus prepared is recommended as an improved substitute for mercurial ointment, or in fact for mercury in any other form. We are not aware that mercurial flannel has been either made or tried in this country; although we have heard of mercurial ointment being placed in the foot of the stockings, as a means of introducing mercury into the system without the trouble of rubbing it in.

